# Socioemotional and Behavioral Problems of Grandchildren Raised by Grandparents: The Role of Grandparent–Grandchild Relational Closeness and Conflict

**DOI:** 10.3390/children10101623

**Published:** 2023-09-29

**Authors:** Yanfeng Xu, Theresa M. Harrison

**Affiliations:** 1 College of Social Work, University of South Carolina, Columbia, SC 29208, USA; 2 Carolina Family Engagement Center, College of Education, University of South Carolina, Columbia, SC 29208, USA; harri642@mailbox.sc.edu

**Keywords:** custodial grandparents, grandparent–grandchild relational closeness and conflict, socioemotional and behavioral well-being, custodial grandchildren

## Abstract

This study examined the associations of grandparent–grandchild relational closeness and conflict with grandchildren’s socioemotional and behavioral problems, including emotional symptoms, conduct problems, hyperactivity, peer problems, and abnormal prosocial behaviors. We analyzed primary cross-sectional survey data collected from custodial grandparents in the United States using logistic regression models. The results indicated that grandparent–grandchild relational closeness was significantly associated with lower odds of custodial grandchildren having emotional symptoms, conduct problems, peer problems, and abnormal prosocial behaviors, whereas grandparent–grandchild relational conflict was significantly associated with higher odds of emotional symptoms, conduct problems, hyperactivity, peer problems, and abnormal prosocial behaviors. Implications for increasing grandparent–grandchild relational closeness and decreasing relational conflicts among grandparent-headed families are discussed, which might improve grandchildren’s socioemotional and behavioral well-being.

## 1. Introduction

In the United States, nearly six million children live with their grandparents for various reasons, such as child maltreatment, parental substance abuse, domestic violence, incarceration, and military deployment [1,2]. Due to COVID-19, an increased number of children lost their parents, and grandparents stepped in to care for them [3]. In the United States, the formation of grandfamilies is influenced by macrosystems (e.g., welfare policies, cultural traditions) and microsystems (e.g., birth parents’ divorce, parental substance abuse, and domestic violence; [4]). Grandparents sometimes assume caregiving responsibilities on short notice due to an emergency placement by a social service agency [5]. Grandfamilies must learn to adapt to these life changes by adjusting their role, routines, and family dynamics [6]. Grandfamilies provide caregiving either formally (i.e., grandparents receive legal custodial status, foster care, or adoption) or informally (i.e., private arrangement; [7]). Grandparent-headed households are the most likely to be in poverty and more likely to receive public assistance and are at risk of living in inadequate housing conditions [8]. Social isolation, stress, poor health, and financial strain are issues that many grandparents raising grandchildren often face [9,10]. Regarding the stability of grandfamilies, children in the care of grandparents compared to other types of out-of-home care have greater stability [11].

In 2018, the Supporting Grandparents Raising Grandchildren Act was passed by the U.S. Congress and called for establishing a federal advisory council to make recommendations that would help grandparents meet the needs of children in their care and maintain their own emotional well-being and mental and physical health. As such, grandparents raising grandchildren has become even more common; thus, further understanding of the well-being of custodial grandchildren is needed.

### 1.1. Custodial Grandchildren’s Socioemotional and Behavioral Problems

An estimated 21% to 33% of grandchildren raised by grandparents (i.e., custodial grandchildren) experience socioemotional or behavioral problems [12]. These co-occurring problems are usually associated with negative outcomes, such as poor academic performance and family functioning problems [13,14]. These socioemotional and behavioral problems may include emotional problems, conduct problems, hyperactivity, peer problems, and abnormal prosocial behaviors [15].

Given the significant impact of child socioemotional and behavioral problems on child development, it is important to unpack the risk and protective factors associated with custodial grandchildren’s socioemotional and behavioral outcomes. A recent systematic review summarized various factors at both the grandchild and grandparent levels associated with custodial grandchildren’s socioemotional and behavioral well-being [16]. At the custodial grandchild level, some known predictors linked with their socioemotional and behavioral outcomes include custodial grandchildren’s race, gender, age, prior traumatic experiences, social support, and self-esteem [17,18,19,20,21]. 

More factors were found at the grandparent level that contributed to custodial grandchildren’s socioemotional and behavioral outcomes. As custodial grandchildren’s primary caregivers, grandparents significantly contribute to grandchildren’s well-being. For instance, custodial grandparents’ education, household income, marital stress, coping resources, and family function can affect grandchildren’s behaviors [22,23]. Furthermore, custodial grandparents’ parenting styles [22] and mental health status (e.g., depression; [13,22,23]) also can affect grandchildren’s socioemotional and behavioral outcomes.

Although some factors associated with grandchildren’s socioemotional and behavioral well-being are known, very limited research has examined the role of the grandparent–grandchild relationship in determining custodial grandchildren’s socioemotional and behavioral problems. Thus, this study seeks to examine the association between the grandparent–grandchild relationship and grandchildren’s socioemotional and behavioral problems, including emotional problems, conduct problems, hyperactivity, peer problems, and abnormal prosocial behaviors. The results of this study provide important implications with a focus on improving the relationship between grandparents and grandchildren, which may help decrease custodial grandchildren’s socioemotional and behavioral problems.

### 1.2. Attachment Theory

Attachment theory provides a theoretical foundation for understanding the impact of family relationships, particularly the relationship between grandparents and grandchildren, on custodial grandchildren’s socioemotional and behavioral problems. Attachment is developed in the first few years of childhood, and children may have more than one attachment figure, like parents or grandparents [24]. The key principles of attachment theory include: (1) if the caregiver is nurturing, the child will achieve a secure attachment. This is demonstrated through proximity-seeking (i.e., protest at separation); (2) secure attachment extends beyond childhood into the social competence exhibited as an adult, and the child uses an attachment figure as a secure base to explore the world (i.e., strangers and objects); and (3) the child uses an attachment figure to seek comfort in times of stress [25,26]. Related to attachment, the profound impact of the positive and secure relationship between children and primary caregivers on children’s lives is well acknowledged. When children experience supportive and warm relationships during their early childhood, they develop a sense of security [27]. This secure attachment further develops into expectations of trustworthiness, which children internalize and take with them throughout their lives. On the other hand, negative attachment experiences in close relationships between caregivers and children can lead to mistrust and hurt future relationships [28]. Overall, attachment theory has been widely used to understand the importance of close and warm relationships in child development.

In the context of grandparenting, grandchildren who are separated from their biological parents have their attachment bonds with their biological parents disrupted. Separating from biological parents may be related to adverse outcomes for children, including mental health problems [29]. However, living with grandparents may rebuild their attachment relationships by allowing them to seek comfort from grandparents when they feel distressed. For some grandchildren, grandparents consistently serve as a source of emotional support when they have not received this support from their parents since they were born. However, for other grandchildren who may have only recently transitioned to the grandparent’s care, it may be unclear whether their attachment to their grandparents is secure or insecure [30]. Disruptions in attachment, whether temporary or permanent, result in cumulative negative effects on the grandchild’s development [24]. Thus, it is important to unpack the relationship between grandparents and grandchildren. Having a close relationship with custodial grandparents may create a secure attachment relationship that fosters grandchildren’s healthy socioemotional and behavioral development.

### 1.3. Grandparent–Grandchild Relationship

Relationships are patterns of interactions, expectations, beliefs, and effects organized at an abstract level that captures observable behaviors [31]. Relationships between grandparents and grandchildren are influenced by many factors, including sociohistorical (e.g., economic pressures, technological changes), cultural (e.g., roles and values regarding grandparenting), family structure (e.g., marital status, custodial grandparenting), and individual differences (e.g., health, gender; [32]). Characteristics of a secure attachment relationship are keeping track of a person, using that person as a secure base from which to explore, being comforted by that person, and being attuned to facial expressions and emotions [33]. Pianta (1992) suggested that relationships can be assessed by relational closeness and conflict between individuals. Relational closeness includes mutual expressions of warmth and positive affect, while relational conflict can include over-control or under-control and/or having dependent or insecure relationships [33].

Grandparents’ perceptions of their relationships with their grandchildren can serve as an indicator of the quality of the relationship. Due to prior traumatic stress and disrupted attachment with primary caregivers, custodial grandchildren usually have a high level of socioemotional and behavioral problems [2]. Custodial grandchildren who struggle with attachment challenges, along with their existing socioemotional and behavioral problems, may have difficulty maintaining close relationships with their grandparents. Because of generational differences and complex family dynamics, grandchildren and grandparents may encounter daily conflicts with each other when they live together. Increased family conflicts may further exacerbate grandchildren’s existing socioemotional and behavioral problems, which has been validated among parent-headed households [34,35].

When grandchildren are placed in grandparent-headed households, having a close grandparent–grandchild relationship is essential and may be a therapeutic means for improving and correcting grandchildren’s socioemotional and behavioral problems and preventing the development of future adverse outcomes [36]. Although there is increasing evidence of the association between family relationships and children’s socioemotional and behavioral problems, a paucity of research has examined the effects of grandparent–grandchild relationships on grandchildren’s socioemotional and behavioral problems. To the authors’ best knowledge, no prior studies have examined the impact of grandparent–grandchild relational closeness and conflict on custodial grandchildren’s emotional symptoms, conduct problems, hyperactivity, peer problems, and prosocial behaviors among grandparent-headed families. Only a few studies have focused on the impact of the grandparent–grandchild relationship on other aspects of well-being among grandchildren. For example, a study found that grandparents’ and grandchildren’s perceived relationship quality was associated with grandchildren’s subjective well-being [37]. Similarly, another study indicated that the grandparent–grandchild relationship predicted grandchildren’s social competence [38]. More specifically, closeness between grandparents and grandchildren was positively associated with grandchildren’s social competence, whereas conflict between grandparents and grandchildren was negatively associated with grandchildren’s social competence. Likewise, Hayslip et al. (2019) revealed that relationship quality between grandparents and grandchildren predicted grandchildren’s relational competence in the United States [39]. Poehlmann et al. (2008) also found that poor family relationships were associated with more behavioral problems among grandchildren in a sample of 79 grandparent-headed families in the United States [24]. In particular, custodial grandchildren with less responsive grandparents were more likely to exhibit aggression, conduct, and hyperactivity issues. Likewise, Pittman et al. (2022) suggested that the relationship quality between grandparents and grandchildren was associated with grandchildren’s psychological outcomes (e.g., depressive symptoms, self-worth, close friendships, and romantic relationships) [40].

Although there is a limited amount of research on the effect of grandparent–grandchild relationships on grandchildren’s socioemotional and behavioral outcomes, existing rich literature on child outcomes as a result of the child–parent relationship (e.g., [34,35,41,42]) provides a solid foundation to understand relational closeness and relational conflict in the context of grandparenting. Generally, relational closeness is a protective factor against children’s socioemotional and behavioral problems. In contrast, relational conflict is a risk factor, but results across studies were slightly different depending on which children’s outcomes were included and how these outcomes were measured [34,35,41,42].

### 1.4. Study Purpose

This study aims to answer the following research question: What are the associations of grandparent–grandchild relational closeness and conflict with grandchildren’s socioemotional and behavioral problems, including emotional symptoms, conduct problems, hyperactivity, peer problems, and prosocial behaviors? The research hypotheses were: (1) Grandparent–grandchild relational closeness is associated with custodial grandchildren’s lower odds of emotional symptoms, conduct problems, hyperactivity, peer problems, and abnormal prosocial behaviors; and (2) Grandparent–grandchild relational conflict is associated with custodial grandchildren’s higher odds of emotional symptoms, conduct problems, hyperactivity, peer problems, and abnormal prosocial behaviors.

## 2. Method

### 2.1. Study Design and Data Collection Procedure

In the present study, we analyzed cross-sectional survey data collected from custodial grandparents raising grandchildren in the United States. The data featured two sources: a South Carolina community-based sample (*n* = 71) and a sample from Qualtrics Panels across the United States except for South Carolina (*n* = 216). We recruited grandparents raising grandchildren in South Carolina via various community partners, including state agencies (e.g., Department of Social Services, Department on Aging), local nonprofit organizations serving kinship families, the local foster parent association, schools, and churches, from May 2021 to February 2022. The survey link was shared with our partners via flyers and email to be used in listservs or online newsletters. We also provided some hard copies of the survey to partners. Most survey data in South Carolina were collected electronically, but a handful of surveys were returned in hard copies. In addition to data collected from South Carolina, we recruited other grandparents raising grandchildren across the United States except for South Carolina via Qualtrics Panels from January to February 2022. Qualtrics Panels is an online survey panel that includes millions of U.S. residents who regularly take online surveys [43]. We used a few inclusion criteria to select custodial grandparents who raised grandchildren: (a) served as a primary caregiver for at least one grandchild younger than 18 years old and (b) the grandchildren lived apart from their biological parents in the United States. To ensure we selected eligible custodial grandparents, we further limited the grandparents’ age to 40 years old or older. To eliminate the possibility of recruiting custodial grandparents in South Carolina who had already filled out our survey, we asked participants if they had filled out the South Carolina survey before. Participants were provided a USD 15 e-gift card for completing the survey.

### 2.2. Sample Selection

In the present study, we limited our sample to custodial grandparents who raised grandchildren between ages 4 and 17 years old (*n* = 255) because the measure of the dependent variable, the Strengths and Difficulties Questionnaire, was only applicable to children in this age range.

### 2.3. Measures

#### 2.3.1. Dependent Variables

Dependent variables were grandchildren’s emotional symptoms, conduct problems, hyperactivity, peer problems, and abnormal prosocial behaviors in the past six months. They were measured using the Strengths and Difficulties Questionnaire (SDQ; 25 items; [15]). The response options were 1 = *not true*, 2 = *somewhat true*, and 3 = *certainly true*. The SDQ has two versions for children between 4 and 17 years old. Most items were the same, but for items with slight differences, we adapted item languages in the survey. Each subscale had five items. Sample items are “Often complains of headaches, stomachaches, or sickness” for emotional problems; “Often fights with other children or bullies them” for conduct problems; “Easily distracted, concentration wanders” for hyperactivity; “Gets along better with adults than with other children” for peer problems; and “Considerate of other people’s feelings” for prosocial behaviors. Some items were reverse coded, and summative scores of these items were calculated first, then these behavioral problems were categorized into four categories (close to average, slightly raised, high, very high) according to brief four-band cutoffs (Department of Health and Ageing, n.d.). Prior empirical research has suggested using 90% cutoffs (i.e., a combination of “close to average” and “slightly raised”) of the SDQ to estimate the general magnitude of mental health problems [12,44]. Therefore, we collapsed “close to average” and “slightly raised” to normal or borderline, and “high” and “very high” to abnormal [45], ending up with five dummy coded dependent variables (1 = *abnormal* and 0 = *normal or borderline*). The reliabilities of emotional symptoms, conduct problems, hyperactivity, peer problems, and prosocial behaviors subscales were 0.82, 0.81, 0.87, 0.65, and 0.79, respectively, in the current sample.

#### 2.3.2. Independent Variables: Grandchild–Grandparent Relational Closeness and Conflict

The independent variables, grandchild–grandparent relational closeness and conflict, were measured using an adapted version of the Child–Parent Relationship Scale (15 items) in the past six months, including relational closeness (seven items) and conflict (eight items) subscales [33]. We modified “child” to “grandchild” to fit the context of this study. The response options ranged from 1 (*definitely does not apply*) to 5 (*definitely applies*). Items included “I share an affectionate, warm relationship with my grandchild,” “My grandchild openly shares his or her feelings and experiences with me,” and “My grandchild remains angry or is resistant after being disciplined.” Summative scores of relational closeness and conflict were used in the present study, and the reliabilities of closeness and conflict were 0.83 and 0.90, respectively.

#### 2.3.3. Control Variables

Control variables at both grandchild and grandparent levels were included in the models. These variables included the grandchild’s race and ethnicity (0 = *White*, 1 = *Black*, and 2 = *other*, including American Indian or Alaska Native, Asian or Asian American, Hispanic or Latinx, and more than one race), age (measured using years), gender (1 = *female* and 0 = *male*), and disability status (1 = *yes* and 0 = *no*). Furthermore, we controlled for grandparents’ characteristics, including race and ethnicity (0 = *White*, 1 = *Black*, and 2 = *other*, including American Indian or Alaska Native, Asian or Asian American, Hispanic or Latinx, and more than one race), gender (1 = *female* and 0 = *male*), age (measured using years), and marital status (1 = *married* and 0 = *other*, including divorced, separated, single, widowed, and other). We also controlled for grandparents’ parenting stress, financial stress, and depression. Parenting stress was measured using one item: “I feel my parenting stress has been increased since the COVID-19 pandemic” (1 = *strongly disagree* to 5 = *strongly agree*).

Grandparent financial stress was measured using the Consumer Financial Protection Bureau Financial Well-Being Scale, including three items: (a) “Because of my money situation, I feel like I will never have the things I want in life”; (b) “I am just getting by financially”; and (c) “I am concerned that the money I have or will save won’t last long” (Consumer Financial Protection Bureau, n.d.). All three items were reverse coded, and the response options ranged from 1 (*completely*) to 5 (*not at all*). An average score was used in the current study, and the reliability was 0.84 in the present sample. Grandparent depression was measured using a shortened version (10 items; [46]) of the Center for Epidemiologic Studies Depression Scale. The scale had four response options (0 = rarely or none of the time (less than 1 day) to 4 = *most of the time (5–7 days)*). A summative score was used, and the reliability of this scale in the present sample was 0.91. Due to the differences in our data collection methods, we also controlled for the data source (1 = *Data collected from Qualtrics Panels* and 0 = *Data collected from South Carolina*).

### 2.4. Strategies to Increase Data Integrity

Our survey was initially attacked by survey bots (i.e., the survey was filled out by computer programs with random responses), but we used a variety of strategies to prevent survey bots and ensure data integrity. These strategies included adding open-ended questions, using inattentional checks, asking identical questions at different points of the survey, embedding reCAPTCHA, and examining survey response patterns (see more details in [47]).

### 2.5. Data Analysis

Data cleaning, descriptive analyses, and logistic regression were conducted using Stata 16.0. All variables except for grandparent race (missing: 1.57%) and financial stress (missing: 0.39%) had no missing data. Grandchildren’s emotional symptoms, conduct problems, hyperactivity, peer problems, and prosocial behaviors were not normally distributed, which violated the assumption of linear regression models. Thus, we used 90% cutoffs of the SDQ to dichotomize emotional symptoms, conduct problems, hyperactivity, peer problems, and prosocial behaviors and conducted logistic regression models. Assumptions of logistic regression were examined, and no violation of these assumptions was identified in the present study. This study was approved by the affiliated university’s institutional review board.

## 3. Results

### 3.1. Preliminary Results

Given the different methods in collecting our data, we conducted a comparison between data collected from South Carolina and Qualtrics Panels using bivariate analyses (i.e., *t*-test, chi-square test). Results indicated significant differences in custodial grandchildren’s conduct problems (χ^2^ = 6.82, *p* = 0.009; South Carolina (SC): 27.0%, Qualtrics: 13.4%) and abnormal prosocial behaviors (χ^2^ = 5.96, *p* = 0.015; SC: 42.3%, Qualtrics: 26.7%), relational conflicts in their grandparent–grandchild relationship (*t* = 2.94, *p* = 0.004; SC: *M* = 18.82; Qualtrics: *M* = 16.01), grandchildren’s race and ethnicity (χ^2^ = 36.82, *p* < 0.001; SC: 43.66% White, 52.11% Black, 4.23% other; Qualtrics: 63.43% White, 17.13% Black, 19.44% other), grandparents’ age (*t* = 5.5, *p* < 0.001; SC: *M* = 60.15, Qualtrics: *M* = 53.50), and grandparents’ race and ethnicity (χ^2^ = 48.55, *p* < 0.001; SC: 41.79% White, 56.72% Black, 1.49% other; Qualtrics: 72.22% White, 15.28% Black, 12.50% other). In other words, custodial grandchildren in South Carolina had more conduct and prosocial behaviors and experienced more relational conflicts with their custodial grandparents than those in the Qualtrics Panels. Regarding the grandchildren’s race and ethnicity, more Black custodial grandchildren and grandparents were identified in the South Carolina sample, and they were older on average than their counterparts from the Qualtrics Panels.

### 3.2. Sample Characteristics

The present study’s sample characteristics are presented in Table 1. In terms of grandchildren’s characteristics, 60% (*n* = 151) were White, followed by Black (24.31%; *n* = 62) and other (15.69%; *n* = 40). The average age of grandchildren was 9.49, ranging from 4 to 17 years old. Half (50.20%; *n* = 128) were boys, and a small proportion (10.20%; *n* = 26) had disabilities. Regarding custodial grandchildren’s socioemotional and behavioral problems, 18.43% (*n* = 47) had abnormal emotional symptoms, 17.65% (*n* = 45) had abnormal conduct problems, 12.55% (*n* = 32) had abnormal hyperactivity, 24.31% (*n* = 62) had abnormal peer problems, and 29.41% (*n* = 75) had abnormal prosocial behaviors. Custodial grandparents reported a high level of relational closeness (30.84 out of 35) and a low level of conflict (16.82 out of 38) with their grandchild.

Many grandparents were White (*n* = 168; 66.93%), followed by Black (*n* = 58; 23.11%) and other (*n* = 25; 9.96%). About 73% (*n* = 185) of grandparents were women, and slightly half (54.90%) were married. The average time that grandparents cared for grandchildren was 4.43 years, and 36.96% had child welfare agency involvement. Grandparents rated their financial stress as 2.88 out of 5, indicating a relatively high level. Custodial grandparents rated their parenting stress as 3.45 out of 5, indicating an even higher parenting stress level. The grandparents’ depression was 8.31 on a range from 0 to 27. Of note, the cutoff for clinically significant depressive symptoms is 8 [46], suggesting that many of the custodial grandparents in the current sample had a high level of depressive symptoms.

### 3.3. Predictors of Custodial Grandchildren’s Socioemotional and Behavioral Problems

Table 2 presents models assessing custodial grandchildren’s socioemotional and behavioral problems. Results consistently indicated that grandparent–grandchild relational closeness was a significant predictor of all socioemotional and behavioral problems among grandchildren, except for hyperactivity. Further, relational conflict between grandparent and grandchild was a significant predictor of all socioemotional and behavioral problems. Specifically, grandparent–grandchild relational closeness was significantly associated with custodial grandchildren’s lower odds of having emotional symptoms (*OR* = 0.87, 95% CI [0.78, 0.97], *p* = 0.013), conduct problems (*OR* = 0.80, 95% CI [0.67, 0.92], *p* = 0.002), peer problems (*OR* = 0.87, 95% CI [0.78, 0.96], *p* = 0.007), and abnormal prosocial behaviors (*OR* = 0.79, 95% CI [0.70, 0.88], *p* < 0.001), whereas grandparent–grandchild relational conflict was significantly associated with higher odds of having emotional symptoms (*OR* = 1.07, 95% CI [1.00, 1.14], *p* = 0.046), conduct problems (*OR* = 1.28, 95% CI [1.15, 1.42], *p* < 0.001), hyperactivity (*OR* = 1.25, 95% CI [1.13, 1.38], *p* < 0.001), and peer problems (*OR* = 1.11, 95% CI [1.04, 1.18], *p* = 0.003).

Regarding other predictors, grandparent depression (*OR* = 1.13, 95% CI [1.05, 1.21], *p* = 0.001) was associated with higher odds that their grandchild experienced emotional symptoms. Grandchildren of other races and ethnicities, compared to their White counterparts, were more likely to have conduct problems (*OR* = 11.61, 95% CI [1.59, 84.93], *p* = 0.016). Female grandchildren were less likely to have conduct problems (*OR* = 0.17, 95% CI [0.04, 0.76], *p* = 0.020) and hyperactivity (*OR* = 0.32, 95% CI [0.11, 0.95], *p* = 0.041) compared to male grandchildren. Furthermore, compared to grandfathers, grandmothers were more likely to report custodial grandchildren’s conduct problems (*OR* = 3.32, 95% CI [0.63, 17.61], *p* = 0.159) and hyperactivity (*OR* = 10.64, 95% CI [2.01, 56.38], *p* = 0.005). Older grandparents were more likely to report hyperactivity (*OR* = 1.08, 95% CI [1.01, 1.15], *p* = 0.020) than younger grandparents. Grandchildren’s disability status was associated with higher odds of hyperactivity (*OR* = 4.69, 95% CI [1.28, 17.10], *p* = 0.0019) and peer problems (*OR* = 5.69, 95% CI [1.85, 17.40], *p* = 0.002).

In addition, this study found that grandchildren from South Carolina had higher odds of having hyperactivity (*OR* = 5.95, 95% CI [1.24, 28.55], *p* = 0.026), while children from South Carolina had lower odds of abnormal prosocial behaviors (*OR* = 0.33, 95% CI [0.12, 0.93], *p* = 0.035) than their counterparts recruited via Qualtrics Panels.

## 4. Discussion

This study examined the role of relational closeness and conflict between grandparents and grandchildren in influencing custodial grandchildren’s socioemotional and behavioral problems. The results of this study partially confirmed our research hypotheses that having a close relationship between grandparents and grandchildren would be associated with lower odds of custodial grandchildren’s emotional symptoms, conduct problems, peer problems, and abnormal prosocial behaviors, but not hyperactivity. Regarding the second hypothesis, results suggested that grandparent–grandchild relational conflict was associated with the higher odds of having all these problems. These major findings highlight the significant need to improve grandparent–grandchild relationships, which may help decrease grandchildren’s socioemotional and behavioral problems.

The current study further identifies that the relational closeness between the grandparent and grandchild is associated with lower odds of having emotional symptoms, conduct problems, and peer problems. This is aligned with attachment theory, where a positive and close relationship helps build grandchildren’s security. Unexpectedly, relational closeness did not predict grandchildren’s hyperactivity. Hyperactivity is a symptom of attention-deficit/hyperactivity disorder that may cause individuals to run, jump, climb, and move constantly [48]. Genetics plays a role in developing hyperactivity among children, which may explain why the relationship and other socioenvironmental factors in the present study did not significantly predict custodial grandchildren’s hyperactivity [49]. In addition, the present study found the significant contribution of relational conflict in predicting grandchildren’s emotional symptoms, conduct problems, peer problems, and hyperactivity, and a marginally significant association with abnormal prosocial behaviors. Prior literature has indicated that family members may have poor emotion regulation and conflict resolution skills in families with high conflicts, exacerbating children’s behavioral problems [50]. Increased relational conflict likely increases parenting and family stress, and children may internalize the stress, ending up with more socioemotional and behavioral problems [51]. In conclusion, the present study’s findings on the associations of relational closeness and conflict with grandchildren’s socioemotional behavioral outcomes were mostly consistent with findings among parent-headed families (e.g., [34,35,41,42]).

Our study also found other interesting variables that contributed to custodial grandchildren’s socioemotional and behavioral problems. For instance, we identified that grandchildren of other races and ethnicities had more conduct problems than White grandchildren. This finding was similar to a previous result where children of other races and ethnicities had more externalizing problems than White children in a sample of children in kinship care [20]. However, we collapsed a few races and ethnicities (i.e., American Indian or Alaska Native, Asian or Asian American, Hispanic or Latinx, and more than one race) in the “other” type, making the explanation of why children of other races/ethnicity having more problems harder. Not surprisingly, female custodial grandchildren had fewer abnormal conduct problems and hyperactivity than their male counterparts, which has been validated in previous studies (e.g., [2]). Grandparents’ age was positively associated with grandchildren’s hyperactivity, which may indicate that older grandparents perceive grandchildren’s hyperactivity more severely because of their low energy level. Also, grandmothers were more likely to report custodial grandchildren’s conduct problems and hyperactivity than grandfathers, which might be because grandmothers as primary caregivers were more likely to notice their grandchildren’s behavioral problems. It is important to understand whether grandchildren are more likely to exhibit emotional and behavioral problems when cared for by grandmothers due to how grandmothers are conditioned to care for grandchildren or perhaps because grandfathers are ignoring symptoms that may need to be brought to the attention of service providers to better assist the family. Another explanation is that grandmothers were more likely to be single, while grandfathers were more likely to have a partner sharing their grandparenting responsibilities.

Another interesting finding was that custodial grandchildren’s disability status was associated with their abnormal hyperactivity and peer problems. Because hyperactivity is a symptom of attention-deficit/hyperactivity disorder, it is not surprising to find these significant associations. In terms of custodial grandchildren’s peer problems, grandchildren with disabilities are at higher risk of experiencing discrimination and social isolation [52,53], and these experiences could contribute to their problems with peers.

Similar to much of the prior literature, custodial grandparents’ depression was associated with the higher odds of grandchildren’s emotional symptoms, peer problems, and abnormal prosocial behaviors [12,23,54]. Depression hampers positive parenting and the interactions between a caregiver and child [55]. Prior research also has indicated that caregivers with depressive symptoms are more likely to report children’s socioemotional and behavioral problems [56]. This highlights the importance of addressing grandparents’ mental health to improve grandchildren’s socioemotional and behavioral well-being.

It was important to note the impact of COVID-19 on the relationships between grandparents and grandchildren and their influences on grandchildren’s elevated socioemotional and behavioral problems because our survey data were collected from May 2021 to February 2022 in the context of the COVID-19 pandemic. For instance, prior empirical evidence has suggested that the increased tension in the relationship between caregivers and children exacerbated the children’s socioemotional and behavioral problems during COVID-19 [57,58].

### 4.1. Limitations and Directions for Future Research

This study has some limitations that should be noted when interpreting results. First, the current study did not control for some COVID-19-related stressors in the analyses. This is a limitation of our study because we cannot overlook the impact of COVID-19 on key variables of the study. Second, the generalizability of the study is limited due to our sampling strategy and data collection methods. Although this study involved grandparents from across the United States, combining the two samples resulted in almost a fourth of participants being from South Carolina. Additionally, the racial demographic in South Carolina was mostly Black and White grandparents, whereas the survey via Qualtrics Panels was open to all races and ethnicities; therefore, more Black grandparents were identified in the South Carolina sample. Further, the average age of South Carolina grandparents was older than the Qualtrics sample. These differences might have skewed our findings. Another limitation of our study is that grandparents were the sole source of data. Grandparents assessed grandparent–grandchild relational closeness and conflict and grandchildren’s socioemotional and behavioral problems. Ideally, having more than one data source would reduce the likelihood of bias in data collection. Further, grandparents reported on their relationships with their grandchildren and their grandchildren’s socioemotional and behavioral problems retrospectively (i.e., recalled these in the past six months when they filled out the survey), which might have influenced the accuracy of their assessments. Moreover, the validity of the grandparent–grandchild relational closeness and conflict scales were not examined in the present study. The reliability of the SDQ peer problems subscale was relatively low (0.65). Last, the study was cross-sectional; therefore, causal linkages between grandparent–grandchildren relationships and grandchildren’s socioemotional and behavioral problems could not be determined.

These limitations point out some directions for future research. First, researchers should use a more rigorous sampling strategy to recruit grandparents when feasible. Second, future studies could consider using multiple informants (e.g., grandchildren, teachers) to collect data when feasible, reducing the risk of reporting bias. In particular, incorporating grandchildren’s perspectives to understand their relationships with grandparents would be important. Third, a longitudinal study would lead to a better understanding of the association between the grandparent–grandchild relationship and grandchildren’s socioemotional and behavioral problems over time. In addition, future research can be enhanced by exploring more patterns of grandparent–grandchild relationships and their associations with grandchildren’s socioemotional and behavioral outcomes. For instance, future research can examine factors that lead to negative family relationships between grandparents and grandchildren and their relationships with biological parents. It would be interesting to examine the factors moderating the relationship, such as child age and timing of custodial grandparent household formation. Lastly, future research could examine the construct validity of the grandparent–grandchild relational closeness and conflict scales.

### 4.2. Implications for Practice

Our study has several important implications for practitioners serving grandparents raising grandchildren. First, our results emphasize the importance of improving the relationship between grandparents and grandchildren, which may help decrease custodial grandchildren’s socioemotional and behavioral problems. Due to the potentially severe consequences of deteriorating family relationships on both grandparents and grandchildren, social workers, family counselors, educators, and other professionals in the community who work with grandparent-headed families need to understand the complexities of these familial relationships. In addition to a thorough and shared understanding of the relationships between grandparents and grandchildren, social workers and other professionals who aim to improve grandchildren’s socioemotional and behavioral problems may pay attention to the relational closeness and conflicts between grandparents and grandchildren. To improve the relational closeness between grandparents and grandchildren, providing relationship-focused interventions could be an option. Practitioners and intervention researchers could consider implementing existing evidence-based interventions with grandparent-headed families. For example, it might be beneficial to implement child–parent relationship therapy (CPRT) among grandparent-headed families. CPRT, adapted from Guerney’s filial therapy [33], is intended for parents of children aged 3 to 10 years who experience emotional or behavioral problems [33]. The primary purpose of CPRT is for parents to learn therapeutic ways of responding to their children’s socioemotional and behavioral problems via improving the parent–child relationship [33,59]. Prior research has indicated that CPRT enhances parent–child relationships (e.g., [59]) and reduces children’s problem behaviors. Adapting this intervention to meet the needs of grandparent-headed families may benefit grandchildren and grandparents.

Social workers, school counselors, and teachers should also advocate for more school-based and health care-based screenings for emotional and behavioral problems among custodial grandchildren who are experiencing family conflict to serve as a preventive measure [60]. School counselors and teachers may serve as good partners with custodial grandparents to monitor children’s behaviors. According to our results, screenings should focus on custodial grandchildren of other races and ethnicities, male and older grandchildren, and grandchildren with disabilities. Furthermore, our study also identified that grandparents with depressive symptoms were more likely to report grandchildren’s emotional symptoms, peer problems, and abnormal prosocial behaviors, which is consistent with prior research (e.g., [56]). Therefore, it is important for them to have access to mental health services and other informal emotional support resources, such as online or face-to-face support groups.

## 5. Conclusions

The current study examined the associations of grandparent–grandchild relational closeness and conflict with custodial grandchildren’s socioemotional and behavioral problems. The findings show that relational closeness was associated with the lower odds of emotional symptoms, conduct problems, peer problems, and abnormal prosocial behaviors, whereas relational conflict was linked to the higher odds of emotional symptoms, conduct problems, peer problems, and hyperactivity among grandchildren. These findings provide implications for social workers to improve relational closeness and decrease relational conflict between grandparents and grandchildren, which may decrease custodial grandchildren’s socioemotional and behavioral problems.

## Figures and Tables

**Table 1:** Characteristics of grandparents and grandchildren (N = 255).

Variable	N	M (SD)/%	Range
**Dependent variables**			
Emotional symptoms			
Normal/borderline	208	81.57%	
Abnormal	47	18.43%	
Conduct problems			
Normal/borderline	210	82.35%	
Abnormal	45	17.65%	
Hyperactivity			
Normal/borderline	223	87.45%	
Abnormal	32	12.55%	
Peer problems			
Normal/borderline	193	75.69%	
Abnormal	62	24.31%	
Prosocial behavior			
Normal/borderline	180	70.59%	
Abnormal	75	29.41%	
**Independent variable**			
Grandparent–grandchild relational closeness	255	30.84 (4.20)	12–35
Grandparent–grandchild relational conflict	255	16.82 (7.51)	8–38
**Control variables**			
Grandchild race/ethnicity	255		
White	153	60.00%	
Black	62	24.31%	
Other	40	15.69%	
Grandchild age	255	9.49 (3.69)	4–17
Grandchild gender	255		
Male	128	50.20%	
Female	127	49.80%	
Grandchild disability status			
Yes	26	10.20%	
No	229	89.80%	
Grandparent race/ethnicity	251		
White	168	66.93%	
Black	58	23.11%	
Other	25	9.96%	
Grandparent gender			
Male	70	27.45%	
Female	185	72.55%	
Grandparent age	255	56.04 (9.08)	40–77
Grandparent marital status			
Married	140	54.90%	
Other	115	45.10%	
Grandparent financial stress	254	2.88 (1.18)	1–5
Grandparent parenting stress	255	3.45 (1.26)	1–5
Grandparent depression	255	8.31 (7.06)	0–27
Data Source			
South Carolina data	71	24.74%	
Qualtrics Panels data	216	75.26%	

**Table 2:** Predictors of grandchildren’s abnormal socioemotional and behavioral problems (N = 250).

	Emotional Symptoms			Conduct Problems			Hyperactivity			Peer Problems			Abnormal Prosocial Behaviors		
	OR	*p*	[95% CI]	OR	*p*	[95% CI]	OR	*p*	[95% CI]	OR	*p*	[95% CI]	OR	*p*	[95% CI]
Grandchild age	0.96	0.581	[0.84, 1.10]	1.03	0.738	[0.85, 1.25]	0.86	0.054	[0.74, 1.00]	1.03	0.623	[0.92, 1.16]	1.05	0.411	[0.94, 1.17]
Grandchild race (ref. White)															
Black	0.77	0.833	[0.06, 9.18]	9.81	0.230	[0.24, 408.33]	0.50	0.536	[0.06, 4.50]	2.46	0.485	[0.20, 30.85]	1.30	0.800	[0.17, 9.93]
Other	0.90	0.897	[0.20, 4.15]	11.61	0.016	[1.59, 84.93]	2.23	0.278	[0.52, 9.47]	0.33	0.207	[0.06, 1.85]	0.89	0.862	[0.23, 3.40]
Grandchild gender (ref. male)	0.84	0.706	[0.35, 2.05]	0.17	0.020	[0.04, 0.76]	0.32	0.041	[0.11, 0.95]	0.78	0.544	[0.35, 1.74]	0.53	0.095	[0.25, 1.12]
Grandchild disability status	1.91	0.706	[0.35, 2.05]	0.65	0.686	[0.08, 5.27]	4.69	0.019	[1.28, 17.10]	5.69	0.002	[1.85, 17.40]	1.66	0.342	[0.58, 4.75]
Grandparent age	0.98	0.376	[0.92, 1.03]	0.94	0.147	[0.85, 1.02]	1.08	0.020	[1.01, 1.15]	0.96	0136	[0.92, 1.01]	0.99	0.679	[0.95, 1.04]
Grandparent race (ref. White)															
Black	0.88	0.924	[0.62, 12.55]	0.22	0.450	[0.01, 11.11]	4.69	0.215	[0.41, 53.88]	0.57	0.682	[0.04, 8.14]	0.75	0.792	[0.09, 6.43]
Other	1.46	0.687	[0.23, 9.24]	0.27	0.320	[0.02, 3.63]	0.93	0.936	[0.14, 6.05]	3.22	0.231	[0.47, 21.82]	1.08	0.925	[0.22, 5.27]
Grandparent gender (ref. male)	1.68	0.325	[0.60, 4.71]	3.32	0.159	[0.63, 17.61]	10.64	0.005	[2.01, 56.38]	1.17	0.735	[0.47, 2.93]	1.84	0.159	[0.79, 4.30]
Grandparent marital status (ref. other)	1.37	0.474	[0.58, 3.21]	0.65	0.501	[0.19, 2.24]	0.97	0.960	[0.35, 2.68]	0.53	0.113	[0.24, 1.16]	0.92	0.827	[0.45, 1.88]
Grandparent financial stress	0.96	0.891	[0.58, 1.60]	1.32	0.454	[0.64, 2.73]	1.19	0.524	[0.70, 2.01]	0.82	0.377	[0.54, 1.27]	0.79	0.241	[0.52, 1.18]
Grandparent parenting stress	1.11	0.636	[0.73, 1.67]	0.89	0.709	[0.47, 1.67]	1.109	0.697	[0.68, 1.80]	1.24	0.251	[0.86, 1.80]	0.97	0.869	[0.70, 1.36]
Grandparent depression	**1.13**	**0.001**	[1.05, 1.21]	1.05	0.350	[0.95, 1.17]	1.02	0.557	[0.94, 1.11]	1.08	0.035	[1.01, 1.15]	1.09	0.009	[1.02, 1.16]
Grandparent–grandchild relational closeness	**0.87**	**0.013**	[0.78, 0.97]	0.80	0.002	[0.67, 0.92]	1.08	0.309	[0.93, 1.25]	0.87	0.007	[0.78, 0.96]	0.79	<0.001	[0.70, 0.88]
Grandparent–grandchild relational conflict	**1.07**	**0.046**	[1.00, 1.14]	1.28	<0.001	[1.15, 1.42]	1.25	<0.001	[1.13, 1.38]	1.11	0.003	[1.04, 1.18]	1.06	0.078	[0.99, 1.12]
Data source (ref: SC data)	0.50	0.289	[0.14, 1.80]	0.35	0.271	[0.06, 2.26]	5.95	0.026	[1.24, 28.55]	1.10	0.871	[0.34, 3.57]	0.33	0.035	[0.12, 0.93]
LR chi^2^	84.52			142.86			68.14			99.69			94.98		
Prob > chi^2^	<0.001			<0.001			<0.001			<0.001			<0.001		
Pseudo R^2^	0.3541			0.6312			0.3636			0.3560			0.3145		

## Data Availability

Data available on request due to restrictions (e.g., privacy or ethical).
